# Expression of Serum microRNAs is Altered During Acute Graft-versus-Host Disease

**DOI:** 10.3389/fimmu.2017.00308

**Published:** 2017-03-24

**Authors:** Rachel E. Crossland, Jean Norden, Mateja Kralj Juric, Kile Green, Kim F. Pearce, Clare Lendrem, Hildegard T. Greinix, Anne M. Dickinson

**Affiliations:** ^1^Haematological Sciences, Medical School, Newcastle University, Newcastle upon Tyne, UK; ^2^Department of Internal Medicine I, Medical University of Vienna, Vienna, Austria; ^3^Division of Hematology, Medical University of Graz, Graz, Austria

**Keywords:** graft-versus-host disease, microRNA, NanoString, biomarkers, hematopoietic stem cell transplantation

## Abstract

Acute graft-versus-host disease (aGvHD) is the most frequent and serious complication following hematopoietic stem cell transplantation (HSCT), with a high mortality rate. A clearer understanding of the molecular pathogenesis may allow for improved therapeutic options or guide personalized prophylactic protocols. Circulating microRNAs are expressed in body fluids and have recently been associated with the etiology of aGvHD, but global expression profiling in a HSCT setting is lacking. This study profiled expression of *n* = 799 mature microRNAs in patient serum, using the NanoString platform, to identify microRNAs that showed altered expression at aGvHD diagnosis. Selected microRNAs (*n* = 10) were replicated in independent cohorts of serum samples taken at aGvHD diagnosis (*n* = 42) and prior to disease onset (day 14 post-HSCT, *n* = 47) to assess their prognostic potential. Sera from patients without aGvHD were used as controls. Differential microRNAs were investigated *in silico* for predicted networks and mRNA targets. Expression analysis identified 61 microRNAs that were differentially expressed at aGvHD diagnosis. miR-146a (*p* = 0.03), miR-30b-5p (*p* = 0.007), miR-374-5p (*p* = 0.02), miR-181a (*p* = 0.03), miR-20a (*p* = 0.03), and miR-15a (*p* = 0.03) were significantly verified in an independent cohort (*n* = 42). miR-146a (*p* = 0.01), miR-20a (*p* = 0.03), miR-18 (*p* = 0.03), miR-19a (*p* = 0.03), miR-19b (*p* = 0.01), and miR-451 (*p* = 0.01) were differentially expressed 14 days post-HSCT in patients who later developed aGvHD (*n* = 47). High miR-19b expression was associated with improved overall survival (OS) (*p* = 0.008), whereas high miR-20a and miR-30b-5p were associated with lower rates of non-relapse mortality (*p* = 0.05 and *p* = 0.008) and improved OS (*p* = 0.016 and *p* = 0.021). Pathway analysis associated the candidate microRNAs with hematological and inflammatory disease. Circulating biofluid microRNAs show altered expression at aGvHD onset and have the capacity to act as prognostic and diagnostic biomarkers. Their differential expression in serum suggests a role for circulatory microRNAs in aGvHD pathology, which warrants further investigation.

## Highlights

- Comprehensive profiling identified 61 microRNAs that demonstrate significant differential expression at aGvHD onset.
- Differential microRNAs can also be detected at day 14 post-HSCT, prior to disease onset.
- High expression of miR-20a is associated with improved overall survival and shows promise as a prognostic indicator for risk of developing aGvHD.
- Dysregulated microRNAs are predicted to target key pathways implicated in aGvHD pathology.

## Introduction

Over 40,000 hematopoietic stem cell transplants (HSCTs) are carried out in Europe each year (1) as curative treatment for a range of hematological diseases, including leukemia and lymphoma. However, up to 40% of HSCT patients will develop graft-versus-host disease (GvHD) in either an acute or a chronic form (2). Acute GvHD (aGvHD) is a serious complication of HSCT and occurs when T cells in the graft elicit an immune response against the host, causing tissue damage in the skin, liver, and gastrointestinal tract (2). It can be life threatening and is the main cause of death post-HSCT. Currently, there are no validated early diagnostic or predictive markers of the disease, which would aid the clinician to either tailor therapy or alter therapeutic regimens on an individual basis to improve outcome.

microRNAs are an interesting novel class of biomarkers, with potential clinical relevance. These small (22–25 nucleotides), non-coding RNAs regulate gene expression by binding to the 3′UTR and repressing translation. Within the last decade, circulating microRNAs have been identified in human plasma and serum (3, 4), where they are resistant to RNase, boiling, changes in pH, extended storage, and freeze–thaw cycles (5). This protection can be attributed to the packaging arrangements used to transport microRNAs to various body systems. microRNAs can be bound by (i) protection proteins (such as nucleophosmin 1 and agonaute 2) (6–8), (ii) lipid or lipoprotein complexes (including high-density lipoprotein and low-density lipoprotein), or (iii) encapsulated into extracellular vesicles such as exosomes (9, 10). Together, these protein and vesicle chaperones allow selective export of microRNAs, protecting them within the extracellular environment (6).

Recently, microRNAs have gained added attention for their potential as biomarkers within circulating biofluids as they are robust, they can be detected by defined and clinically translatable technologies, and additionally, biofluids can be collected in quantity using non-invasive methods. Despite previous interest focusing on circulating protein biomarkers (11), microRNAs may offer several advantages as they are lower in complexity, conserved among clinically relevant species, expressed specifically in different tissues or biological stages, and easily measured using common laboratory techniques (12).

Preliminary studies have assessed expression of specific circulating microRNAs in the serum or plasma of patients post-HSCT to identify potential biomarkers for aGvHD incidence, severity, or outcome (13–17), and some investigations have included profiling of an extended microRNA repertoire (13, 17). However, these studies have focused on a subset of microRNAs that can be detected by high-throughput qRT-PCR-based arrays (13, 17) or within specific subsets of patients such as lymphoma patients receiving matched unrelated donor (MUD) allo-HSCT (17).

Thus, comprehensive profiling of the microRNA expression spectrum in relation to aGvHD, which takes into account the heterogeneity of the HSCT clinical setting including underlying disease, conditioning, and prophylaxis, is lacking. Consequently, much work is needed to reproducibly identify microRNAs that are deregulated in aGvHD and validate these findings in independent cohorts, which reflect the diversity in clinical protocols employed by different transplant centers.

This study aimed to comprehensively profile *n* = 799 highly conserved mature human microRNAs in post-HSCT patient serum using NanoString nCounter technology, to explore the spectrum of microRNAs demonstrating differential expression at aGvHD diagnosis. This provides a sensitive, highly multiplexed method for detecting direct digital counts of each microRNA, without the need for reverse transcription or amplification. To illustrate the prominence of microRNAs differentially expressed at aGvHD onset, candidate microRNAs were verified and then further explored for their prognostic potential in an independent cohort.

## Materials and Methods

### Clinical Cohorts and Ethics

microRNA expression analysis was performed for *n* = 12 patients (aGvHD *n* = 6, no aGvHD *n* = 6) undergoing allogeneic HSCT at the Medical University Hospital Vienna between 2010 and 2014 (mean diagnosis 35 days, range 7–62 days) (Table 1). Replication and expansion of NanoString data were performed on two independent cohorts: diagnostic (*n* = 42) (collected at the time of aGvHD diagnosis or equivalent time point for no aGvHD cases, ±3 days) (aGvHD *n* = 24, no aGvHD *n* = 18) (mean diagnosis 29 days, range 14–90 days) or prognostic (*n* = 47) (collected on day 14 (D14) post-HSCT) (aGvHD *n* = 24, no aGvHD *n* = 23) (mean diagnosis 41 days, range 20–100 days) serum samples collected from allogeneic HSCT patients undergoing transplantation between 2008 and 2014 at the Freeman Hospital, Newcastle upon Tyne, UK (Table 2).

**Table 1:** **Clinical details of the NanoString cohort**.

ID	Disease status	Date Tx	Patient age (years)	Patient gender	aGvHD Proph	Days onset aGvHD	aGvHD grading	Sample day (post-HSCT)	Dx	Tx type	Cond
1	aGvHD	23/06/2014	52	F	CSA + MMF	57	Skin II	57	CML	MUD	RIC
2	aGvHD	30/07/2014	33	M	CSA + MMF	62	Skin II	62	AML	MUD	RIC
3	aGvHD	09/05/2014	56	F	CSA + MMF	26	Skin II/GI II	26	MDS	MUD	RIC
4	aGvHD	10/06/2014	58	M	CSA + MMF	23	Skin II	23	MDS	SIB	RIC
5	aGvHD	17/07/2014	51	F	CSA	34	Skin III	34	AML	MUD	MYO
6	aGvHD	14/11/2014	27	M	CSA + MTX	7	Skin II	7	MDS	SIB	RIC
7	No aGvHD	20/01/2010	57	M	CSA + MTX	*N/A*	N/A	57	AML	MUD	RIC
8	No aGvHD	22/03/2013	60	M	CSA + MMF	*N/A*	N/A	31	AML	MUD	RIC
9	No aGvHD	11/04/2013	41	M	CSA + MMF	*N/A*	N/A	40	ALL-T	MUD	RIC
10	No aGvHD	05/07/2013	51	M	CSA + MTX	*N/A*	N/A	45	CML	SIB	MYO
11^a^	No aGvHD	17/01/2014	47	M	CSA + MTX	*N/A*	N/A	38	AML	MUD	MYO
12	No aGvHD	04/04/2014	43	F	CSA + MTX	*N/A*	N/A	40	AML	SIB	RIC

*A total of 12 diagnostic serum samples taken from Vienna patients at aGvHD onset (*n* = 6) or from aGvHD-free controls (*n* = 6) were analyzed for microRNA expression using NanoString*.

*Tx, transplant; Proph, prophylaxis; Dx, diagnosis; Cond, conditioning; F, female; M, male; CSA, cyclosporine A; MMF, mycophenolate mofetil; MTX, methotrexate; GI, gastrointestinal; CMML, chronic myelomonocytic leukemia; AML, acute myeloid leukemia; MDS, myelodysplastic syndrome; ALL-T, T-lineage acute lymphoblastic leukemia; CML, chronic myeloid leukemia; MUD, matched unrelated donor; SIB, sibling donor; RIC, reduced intensity conditioning; MYO, myeloablative conditioning; N/A, not applicable*.

*^a^Sample removed from the final NanoString data analysis due to failed quality control parameters*.

**Table 2:** **Clinical and outcome details of the diagnostic and prognostic cohorts**.

	Diagnostic cohort				Prognostic cohort				Cohort comparison
	All	No GvHD, *N* (%)	aGvHD, *N* (%)	*p* Value	All	No GvHD, *N* (%)	aGvHD, *N* (%)	*p* Value	*p* Value
All	42	18	24		47	23	24		
**Patient gender^a^**									
Male	27 (64)	11 (61)	16 (67)	0.75^a^	23 (49)	10 (43)	13 (54)	0.56^a^	0.20^a^
Female	15 (36)	7 (39)	8 (33)		24 (71)	13 (57)	11 (46)		
**Donor gender^a^**									
Male	32 (76)	14 (78)	18 (75)	1.0^a^	34 (72)	17 (74)	17 (71)	1.0^a^	0.81^a^
Female	10 (24)	4 (22)	6 (25)		13 (28)	6 (26)	7 (29)		
**Mean age (years)^b^**	51	50	52	0.62^b^	49	49	49	0.98^b^	0.82^b^
Range (years)	20–65	26–61	20–65		20–69	24–68	20–69		
**Relationship^a^**									
MUD	36 (86)	14 (78)	22 (92)	0.38^a^	28 (60)	10 (43)	18 (75)	0.04^a^	0.02^a^
SIB	6 (14)	4 (22)	2 (8)		19 (40)	13 (56)	6 (25)		
**Conditioning^a^**									
RIC	35 (83)	15 (83)	20 (83)	1.0^a^	20 (43)	19 (83)	16 (67)	0.32^a^	0.01^a^
Myeloablative	7 (17)	3 (17)	4 (17)		27 (57)	4 (17)	8 (33)		
**Outcome^a^**									
Alive	29 (69)	15 (83)	14 (58)		28 (60)	15 (65)	13 (54)	0.56^a^	0.38^a^
Dead	13 (31)	3 (17)	10 (42)	0.10^a^	19 (40)	8 (35)	11 (47)		

*Clinical details of the diagnostic (*n* = 42) (collected at the time of aGvHD diagnosis ±3 days) and prognostic (*n* = 47) (collected on day 14 post-HSCT) Newcastle cohorts to test for differences between aGvHD and no aGvHD groups and also between the diagnostic and prognostic cohorts. Outcome was evaluated from the date of death or the date last assessed in clinic*.

*p Values were calculated using the Fisher’s exact test^a^ or independent two sample t-test^b^, as appropriate*.

*MUD, matched unrelated donor; SIB, sibling donor; RIC, reduced intensity conditioning; N, number; HSCT, hematopoietic stem cell transplantation; aGvHD, acute graft-versus-host disease*.

All patients consented for sample collection and molecular testing, and the project was approved by the Newcastle and North Tyneside I Research Ethics Committee and the Ethics Committee of the Medical University of Vienna, Austria. The overall clinical aGvHD grade was diagnosed by clinicians in accordance with the NIH consensus and modified Glucksberg criteria (18, 19). All the clinical data were obtained from the EuroTransplantBank database.^1^ Investigations were conducted in accordance with the Helsinki Declaration.

### Serum RNA Isolation

Whole blood samples were collected in 7 ml vacutainers containing no anticoagulant and left to clot, and the supernatant was centrifuged at 500 *g* for 5 min and then stored at −80°C. Total RNA was isolated from 250 μl aliquots using the Norgen Biotek Total RNA kit, following the supplier’s instructions. For NanoString profiling, the RNA isolated from 4 × 250 μl serum aliquots was concentrated to 25 μl by pooling replicate RNA isolations using a NanoString Technologies-approved centrifugation protocol, incorporating Amicon Ultra-0.5 Centrifugal Filter Units (Merck Millipore). All RNA was quantified using the Bioanalyzer and RNA 6000 Pico kit (Agilent), and interassay variation was reported as 7.21%. Variation in RNA recovery between samples was compensated for by the use of NanoString and qRT-PCR endogenous controls.

### NanoString microRNA Profiling

Total RNA was profiled using the nCounter^®^ Human v3.0 miRNA Expression Assay Kit (NanoString Technologies), based on miRBase v21. The code set incorporated 799 mature microRNAs and included 6 positive controls, 8 negative controls, 6 ligation controls, and 5 mRNA housekeeping controls (*ACTB, B2M, GAPDH, RPL19* and *RPLP0*). Starting material comprised 3 μl of concentrated serum RNA. Data normalization was performed using nSolver Analysis Software v2.5 (NanoString Technologies), with code set content normalization to the top 100 microRNAs established using geometric means.

### TaqMan microRNA Analysis

Individual microRNAs were evaluated in independent diagnostic and prognostic cohorts by TaqMan qRT-PCR. Briefly, microRNA and endogenous control [HY3 and U6 (20)] specific cDNA was generated using TaqMan^®^ Assays and the TaqMan^®^ microRNA Reverse Transcription kit (Life Technologies), according to the supplier’s protocol. Each 15 μl reaction incorporated 4 μl total RNA. Quantitative RT-PCR was performed incorporating SensiFast Probe Hi-Rox reagent (Bioline). Each 10 μl reaction incorporated 3.25 μl cDNA, and thermal cycling was performed in triplicate using the 7900HT Real-Time PCR System (Life Technologies), according to manufacturer’s recommended conditions. The interassay and intraassay variation were reported for an example microRNA (miR-15a: interassay = 1.89%, intraassay = 2.26%) for proof of principle of the quantitative performance of qRT-PCR (Table S1 in Supplementary Material). In addition, an example microRNA (miR-146a) was assessed in normal healthy controls (*n* = 3), in triplicate technical repeats, across three consecutive time points (Day 1–3), to demonstrate stability of microRNA expression across repetitive measurements (Figure S1 in Supplementary Material).

### Ingenuity Pathway Analysis

The biological targets of identified microRNAs were investigated using QIAGEN’s Ingenuity^®^ Pathway Analysis (IPA^®^).^2^ Interactions and networks between significant genes and microRNAs were mapped to pathways, regulators, diseases, and functions based on direct/indirect and experimentally validated targets.

### Statistical Analysis

For NanoString analysis, fold change (FC) expression differences between two groups were calculated using nSolver v2.5 (NanoString Technologies) ratio data, based on normalized count data. Further analysis was performed using a pipeline designed by Newcastle University, Haematological Sciences Department. This integrated a number of “R” (R project) statistical packages in the “R” programming language. *p* Values between two groups were generated using a two-tailed *t*-test. Volcano plots were generated using functions within the “ggplot2” (v2.1.0) package, and heatmaps were constructed using “gplots” (v2.17.0) and “RColorBrewer” (v1.1-2), based on an unsupervised clustering approach of the normalized expression counts, with a Euclidean (L2 norm) distance measure and “Complete” as the agglomeration method. Quantitative RT-PCR microRNA expression was assessed using SDS2.4 software (Life Technologies) and analyzed using GraphPad Prism v6.0, SPSS v22.0 and SigmaPlot v12.5. Differences between groups were assessed using the independent or paired *t*-test (two groups) or one-way ANOVA. Receiver operating characteristic (ROC) analysis was performed using disease or survival status as the binary state (classification) variable and marker expression on a continuous scale as the test variable to determine area under the curve (AUC) (SigmaPlot v12.5). The ROC posttest results used a pretest prior-probability of aGvHD of 0.5 and cost ratio of 1.0 (21). Overall survival (OS) was calculated using the time in years from transplant to death or last follow-up. Survival plots were generated using the Kaplan–Meier method, and differences in outcome were assessed for significance using the Log-Rank test (SPSS v22). The threshold to determine dichotomies for microRNA expression (low and high expression) was evaluated by ROC analysis. Cumulative incidence based on the competing risk method, as described by Fine and Gray (22), was used for assessing the association between microRNAs and both relapse and non-relapse mortality (NRM) (23) using “R” package “cmprsk” (competing risks) (24). To test for confounding variables, each clinical variable for which data were available was added to the model (Cox Regression), and the hazard ratio was assessed for >10% difference, after adjusting for each variable. Correlation between microRNA expression levels was determined using Pearson’s correlation with Holm–Bonferroni multiple comparisons adjustment applied (25).

## Results

### Clinical Details of the Cohorts

NanoString microRNA expression profiling was performed on serum samples taken from 12 adult (mean age 48 years, range 27–60 years) allo-HSCT patients transplanted between 2010 and 2014 at the Department of Internal Medicine I, Medical University of Vienna, Austria (Table 1). There were *n* = 8 MUD, *n* = 4 sibling, *n* = 9 reduced intensity, and *n* = 3 myeloablative transplants. There were four female and eight male patients and aGvHD prophylaxis included cyclosporine ± mycophenolate mofetil or methotrexate. Six patients showed clinical symptoms of aGvHD at the time of sample collection (skin II–III and/or gastrointestinal II), with a mean time of onset of 35 days (range 7–62 days post-HSCT), while six were aGvHD free (Table 1).

Verification of NanoString results was performed using a diagnostic cohort (*n* = 42) of serum samples taken from patients at the onset of aGvHD symptoms (mean 29 days, range 14–90 days post-HSCT) who were transplanted at a separate European Institution (Newcastle) (Table 2). As conditioning and prophylaxis regimens can be highly heterogeneous according to the transplant center, replication in an independent cohort is essential to identity microRNAs that are differentially expressed in aGvHD serum, regardless of the transplant protocols. The cohort comprised *n* = 18 (43%) no aGvHD and *n* = 24 (57%) aGvHD (grade II *n* = 22, grade III *n* = 2) HSCT patients transplanted between 2011 and 2014. Clinical presentation included skin only (*n* = 15), skin and intestine (*n* = 2), skin and liver (*n* = 1), and liver only (*n* = 1), while the sites of aGvHD manifestation were unknown for *n* = 5 patients. Transplants included both reduced intensity conditioning (RIC)/myeloablative and MUD/sibling donor (SIB) relationships (Table 2). There was no significant difference in patient gender (*p* = 0.75), donor gender (*p* = 1.0), age (*p* = 0.62), relationship to donor (*p* = 0.38), or conditioning regimen (*p* = 1.0) between those who developed aGvHD vs. no aGvHD (Table 2). Similarly, there was no significant difference in patient gender (*p* = 0.10), age (*p* = 0.31), relationship to donor (*p* = 0.23), or conditioning regimen (*p* = 0.24) between the NanoString (*n* = 12) and verification diagnostic (*n* = 42) cohorts. The presence of aGvHD was associated with clinical outcome in the cohort (NRM *p* = 0.053).

To further explore the biomarker potential of NanoString identified microRNAs, selected microRNAs were also assessed in a prognostic cohort (*n* = 47) of serum samples taken from HSCT patients at D14 posttransplant, from the same center as the diagnostic cohort (Newcastle), prior to the onset of aGvHD (no patients had aGvHD at the time of sampling; mean aGvHD onset 41 days, range 20–100 days post-HSCT). The prognostic cohort comprised *n* = 23 (49%) no aGvHD and *n* = 24 (51%) aGvHD (grade II *n* = 22, grade III *n* = 2) patients transplanted between 2008 and 2013 in Newcastle (Table 2). There was no significant difference in patient gender (*p* = 0.56), donor gender (*p* = 1.0), age (*p* = 0.98), or conditioning regimen (*p* = 0.32) between patients who later developed aGvHD vs. those who remained disease free. There was a significant (*p* = 0.04) baseline difference between aGvHD vs. disease-free patients for transplant relationship: there was a higher percentage of aGvHD patients who were MUD transplanted compared to those who were disease free, and there was also a higher percentage of disease-free patients who were SIB transplanted compared to those who developed aGvHD (Table 2). However, when we adjusted for transplant relationship in a logistic model with aGvHD as the response, the relationship between microRNA and aGvHD was unaffected (data not shown). There was no significant difference in patient gender (*p* = 0.20) or age (*p* = 0.82) between the diagnostic and prognostic cohorts; however, there was a higher percentage of SIB transplants and myeloablative conditioning in the prognostic cohort compared to the diagnostic cohort (Table 2). There was no significant difference in clinical outcome between the diagnostic and prognostic cohorts (*p* = 0.38) (Table 2).

### microRNA Expression Analysis at aGvHD Diagnosis

NanoString results for one sample (patient number 11, no aGvHD) demonstrated poor data normalization and thus did not pass quality control parameters. Subsequently, this sample was excluded from further analysis.

**Figure 1:**
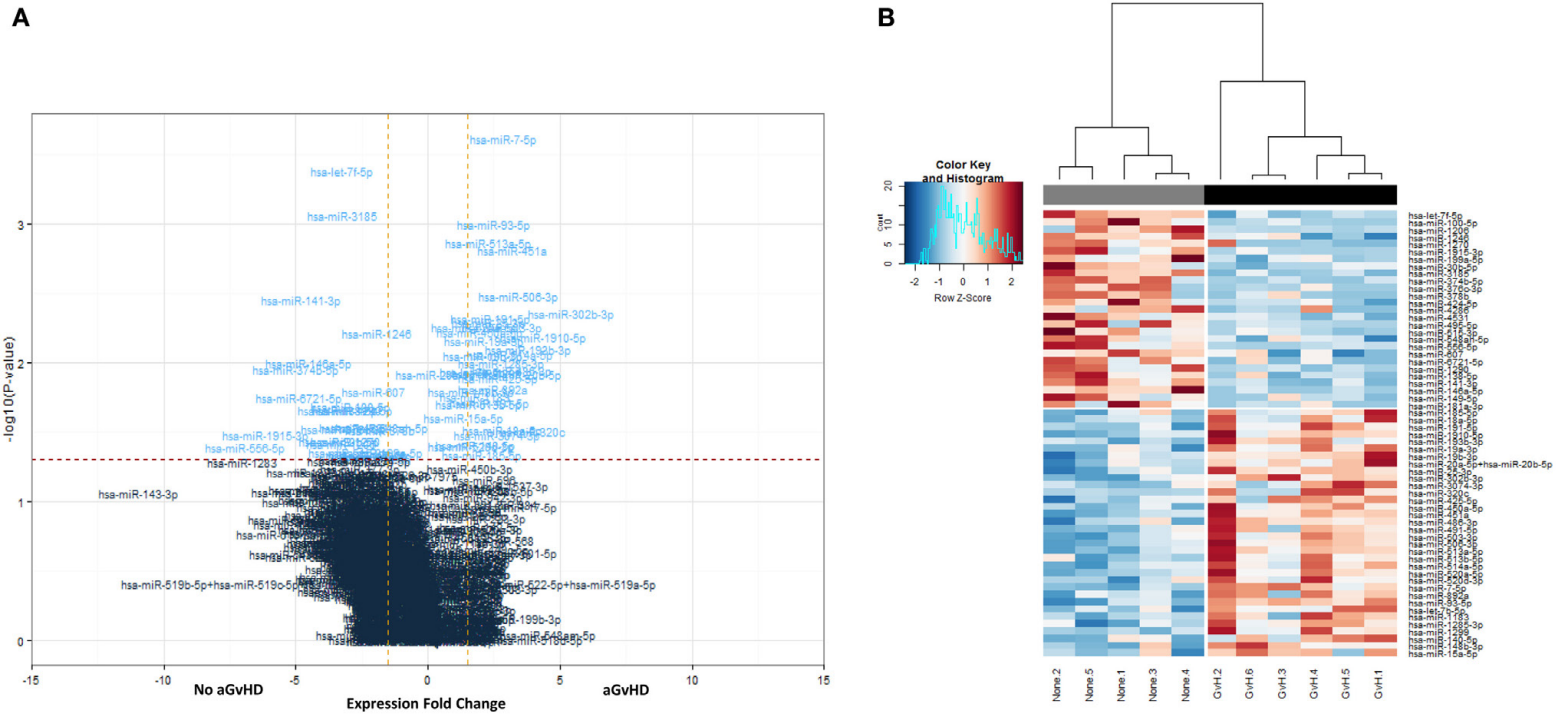
**NanoString-detected fold changes (FCs) in microRNA quantity between acute graft-versus- host disease (aGvHD) vs. no aGvHD patients**. Eleven allohematopoietic stem cell transplantation patient serum samples (six aGvHD and five no aGvHD) were included in the final data set for microRNA expression assessment (*n* = 799) using the NanoString microRNA panel. **(A)** Volcano plot to show the relationship between FC and significance between the two groups. A FC comparison was made comparing aGvHD to no aGvHD. The horizontal dashed line indicates cutoff for significance *p* < 0.05 (−log10 *p* > 1.3) and the vertical lines for FC ≥ 1.5/≤−1.5. **(B)** Heatmap showing hierarchical clustering of significantly differentially expressed microRNAs (*p* < 0.05, *n* = 61), based on normalized digital expression counts, in serum samples between patients with aGvHD vs. no aGvHD. Each column represents an individual patient. Relative expression changes are indicated by the color scale (red: high; blue: low). No aGvHD (none) cases are indicated in gray, while aGvHD cases (GvH) are indicated in black.

All 799 target microRNAs of the code set were detected in *n* = 11 serum samples assessed, and unsupervised hierarchical clustering analysis was able to clearly separate aGvHD and non-aGvHD patients. A total of *n* = 61 microRNAs were significantly differentially expressed in aGvHD (*n* = 6) compared to no aGvHD (*n* = 5), of which *n* = 27 were downregulated (FC −6.94 to −1.75; *p* < 0.01 to *p* = 0.048) in aGvHD, while *n* = 34 were upregulated (FC 1.35–5.41; *p* < 0.01 to *p* = 0.046) (Table S2 in Supplementary Material) (Figures 1A,B).

### Replication of Differential microRNA Expression at aGvHD Diagnosis

To verify the NanoString data, 10 microRNAs (miR-146a, miR-30b-5p, miR-374-5p, miR-20a, miR-15a, miR-181a, miR-18a, miR-19a, miR-19b, and miR-451a) were selected for further assessment in the diagnostic verification cohort (Table 2), based on those with high FC and/or reported in the literature to be implicated in GvHD, T-cell function, or the inflammatory response (13, 26–32).

**Figure 2:**
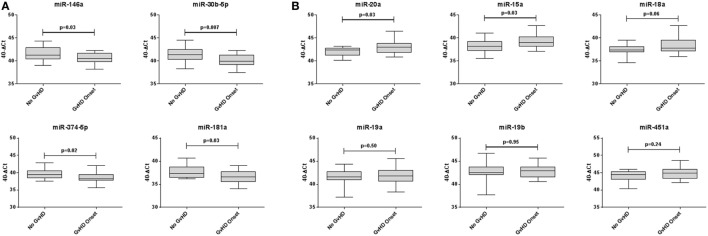
**Differential microRNA expression in the diagnostic verification cohort**. Expression of the candidate microRNAs was assessed by qRT-PCR at the onset of acute graft-versus- host disease (aGvHD) symptoms in serum samples of the diagnostic verification cohort (*n* = 42) and analyzed according to aGvHD incidence. **(A)** Verification of microRNAs that were downregulated at aGvHD onset according to NanoString analysis. **(B)** Verification of microRNAs that were upregulated at aGvHD onset according to NanoString analysis. Box plot whiskers represent minimum to maximum expression, and *p* Values were calculated using the independent two sample *t*-test.

**Figure 3:**
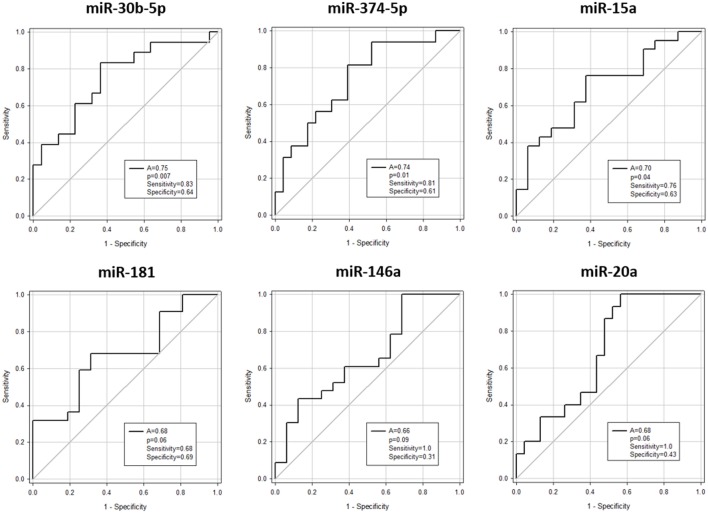
**Association between serum microRNAs and acute graft-versus- host disease (aGvHD) at diagnosis by receiver operating characteristic analysis**. Expression of candidate microRNAs was assessed by qRT-PCR at aGvHD onset in the verification cohort (*n* = 42) and analyzed according to aGvHD incidence. Receiver operating characteristic curves detailing area under the curve (*A*), *p* value, sensitivity, and specificity are shown.

Expression of miR-146a (*p* = 0.03), miR-30b-5p (*p* = 0.007), miR-374-5p (*p* = 0.02), and miR-181a (*p* = 0.03) was significantly downregulated, whereas miR-20a (*p* = 0.03) and miR-15a (*p* = 0.03) were significantly upregulated in aGvHD, in agreement with NanoString data (Figures 2A,B). miR-18a was also upregulated in aGvHD, but not significantly (*p* = 0.06) (Figure 2B). There was no differential expression of miR-19a (*p* = 0.50), miR-19b (*p* = 0.95), or miR-451a (*p* = 0.24) (Figure 2B). miR-30b-5p (AUC = 0.75, *p* = 0.007), miR-374-5p (AUC = 0.74, *p* = 0.01), and miR-15a (AUC = 0.70, *p* = 0.04) had diagnostic utility for aGvHD as assessed by ROC analysis, whereas miR-181 (AUC = 0.68, *p* = 0.06), miR-146a (AUC = 0.66, *p* = 0.09), and miR-20a (AUC = 0.68, *p* = 0.06) were approaching significance (Figure 3). Two-sample *t*-tests were performed to check for baseline differences in microRNA expression between aGvHD and no aGvHD groups for transplant relationship, patient gender, and conditioning (data not shown). Although some of the tests gave borderline-significant results, these were not consistent across cohorts, so it is unlikely that any differences in the microRNAs are caused by clinical factors.

### Correlations between microRNAs at aGvHD Diagnosis

Correlation analysis was performed between all candidate microRNAs assessed in the diagnostic verification cohort. The majority of the microRNAs demonstrated significant positive correlation (Table S3 in Supplementary Material). miR-19b (eight correlations, *p* < 0.01 to *p* = 0.011), miR-15a (eight correlations, *p* < 0.01 to *p* = 0.025), and miR-19a (eight correlations, *p* < 0.01 to *p* = 0.044) were the most highly correlated microRNAs, followed by miR-181 (six correlations, *p* < 0.01 to *p* = 0.044), miR-374-5p (six correlations, *p* < 0.01 to *p* = 0.014), miR-30b-5p (six correlations, *p* < 0.01 to *p* = 0.005), miR-18a (five correlations, *p* < 0.01 to *p* = 0.025), miR-451 (five correlations, *p* < 0.01 to *p* = 0.016), miR-20a (four correlations, *p* < 0.01 to *p* = 0.002), and miR-146a (four correlations, *p* < 0.01 to *p* = 0.001) (Table S3 in Supplementary Material).

### Association between microRNAs Differentially Expressed at aGvHD Diagnosis and Clinical Outcome

**Figure 4:**
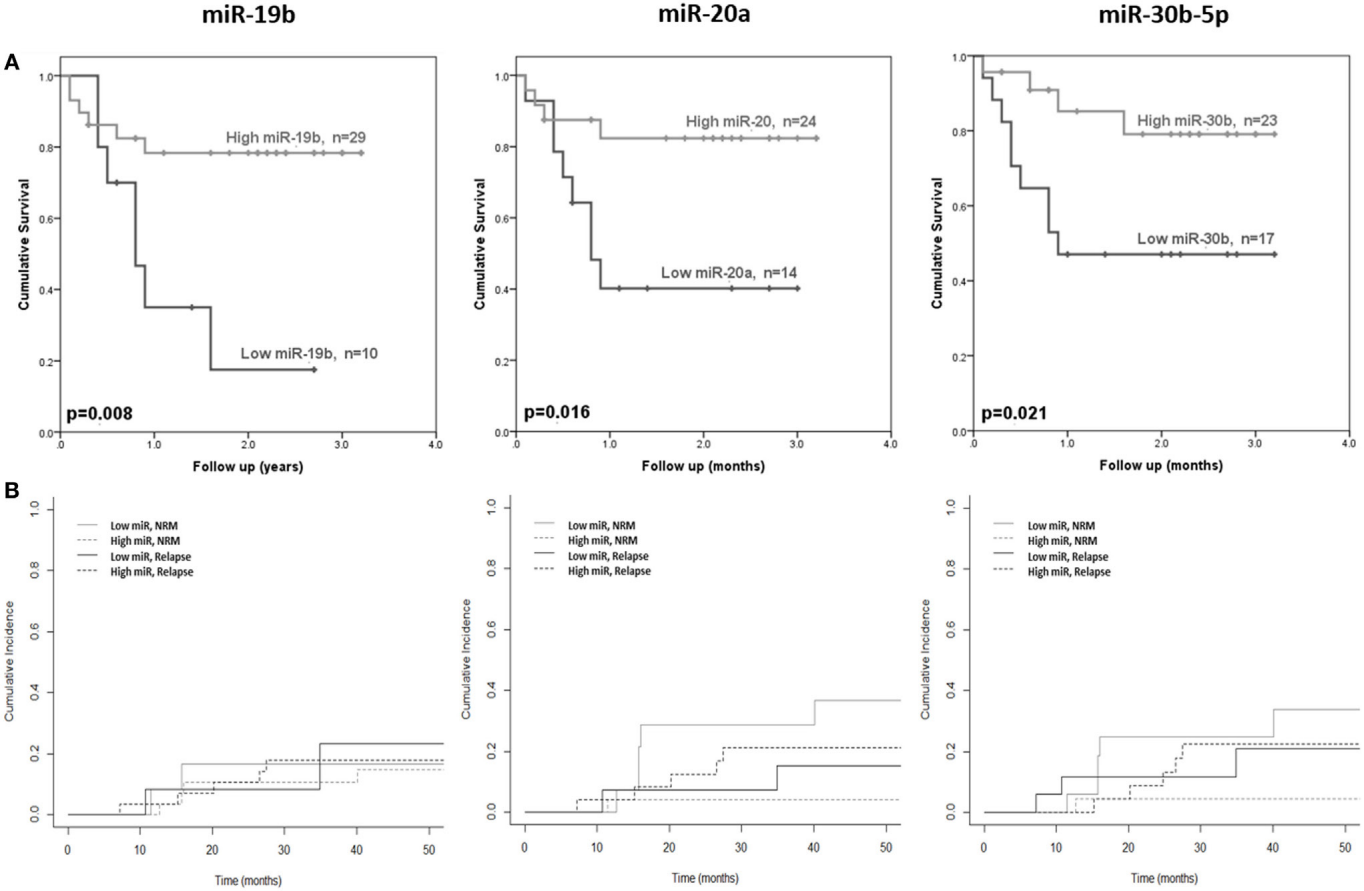
**Association between serum microRNAs and acute graft-versus- host disease (aGvHD) clinical outcome**. Expression of candidate microRNAs was assessed by qRT-PCR at aGvHD onset in the verification cohort (*n* = 42) and analyzed according to outcome. **(A)** Overall survival according to microRNA expression in relation to follow-up from time of transplant to event. *p* Values were calculated using the log-rank test. **(B)** Non-relapse mortality (NRM) according to microRNA expression in relation to time from transplant to event. Dichotomized microRNA expression in relation to relapse and NRM is depicted by dotted and solid black and gray lines, respectively. *p* Values were calculated according to the Fine and Gray competing risk method.

The 10 microRNAs that were selected for analysis in the diagnostic certification cohort were assessed for association with clinical outcome (OS and NRM). High expression of miR-19b was significantly associated with improved OS (*p* = 0.008) (Figure 4A), but showed no association with NRM (*p* = 0.94) (Figure 4B). Elevated expression of miR-20a and miR-30b-5p was significantly associated with improved OS (*p* = 0.016 and *p* = 0.021, respectively) (Figure 4A) and lower NRM (*p* = 0.05 and *p* = 0.008, respectively) (Figure 4B). Statistical modeling to adjust for confounding variables was performed, by adding each clinical variable for which clinical data were available (aGvHD grade, patient age, conditioning regimen, donor relationship, donor gender, and patient gender) to the model as a potential confounding variable. In each case, the hazard ratio after adjusting for each variable did not differ by >10%, indicating a true relationship between expression of the microRNA and NRM.

### Differential microRNA Expression prior to aGvHD Diagnosis

**Figure 5:**
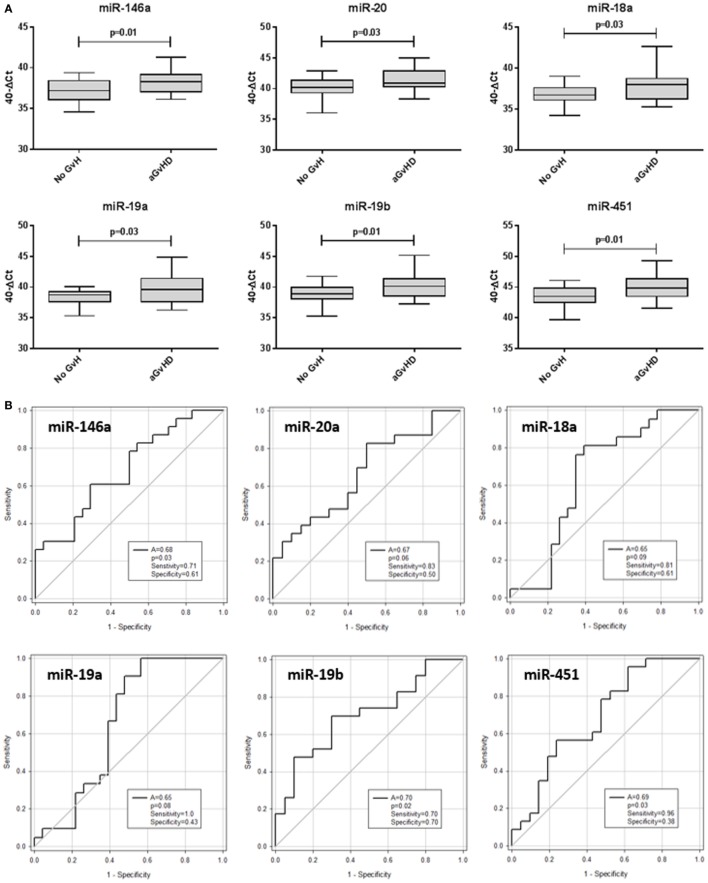
**Differential microRNA expression in the prognostic cohort**. Expression of the candidate microRNAs was assessed by qRT-PCR at day 14 posthematopoietic stem cell transplantation in the validation prognostic cohort (*n* = 47) and analyzed according to acute graft-versus-host disease (aGvHD) incidence. **(A)** microRNA expression according to aGvHD vs. no GvHD. Box plot whiskers represent minimum to maximum expression, and *p* values were calculated using the independent two sample *t*-test. **(B)** Receiver operating characteristic curves for incidence of aGvHD to determine prognostic ability of microRNA expression. The area under the curve (*A*), *p* value, sensitivity, and specificity are shown.

The same 10 microRNAs were also assessed at D14 post-HSCT, prior to disease onset, in an independent prognostic cohort (Table 2) to investigate their prognostic biomarker potential. miR-146a (*p* = 0.01), miR-20a (*p* = 0.03), miR-18a (*p* = 0.03), miR-19a (*p* = 0.03), miR-19b (*p* = 0.01), and miR-451 (*p* = 0.01) were expressed at significantly higher levels in patients who went on to develop aGvHD vs. no aGvHD (Figure 5A). In ROC analysis, miR-146a (*A* = 0.68, *p* = 0.03), miR-19b (*A* = 0.70, *p* = 0.02), and miR-451 (*A* = 0.69, *p* = 0.03) had diagnostic ability with regards to aGvHD incidence, whereas miR-18a (*A* = 0.65, *p* = 0.09), miR-19a (*A* = 0.65, *p* = 0.08), and miR-20 (*A* = 0.67, *p* = 0.06) were approaching significance (Figure 5B). microRNA expression at D14 was assessed for prediction of HSCT outcome; however, no microRNAs were significantly associated with OS or NRM (*p* > 0.05, data not shown).

### Comparison of microRNA Expression between Prognostic and Diagnostic Time Points

**Figure 6:**
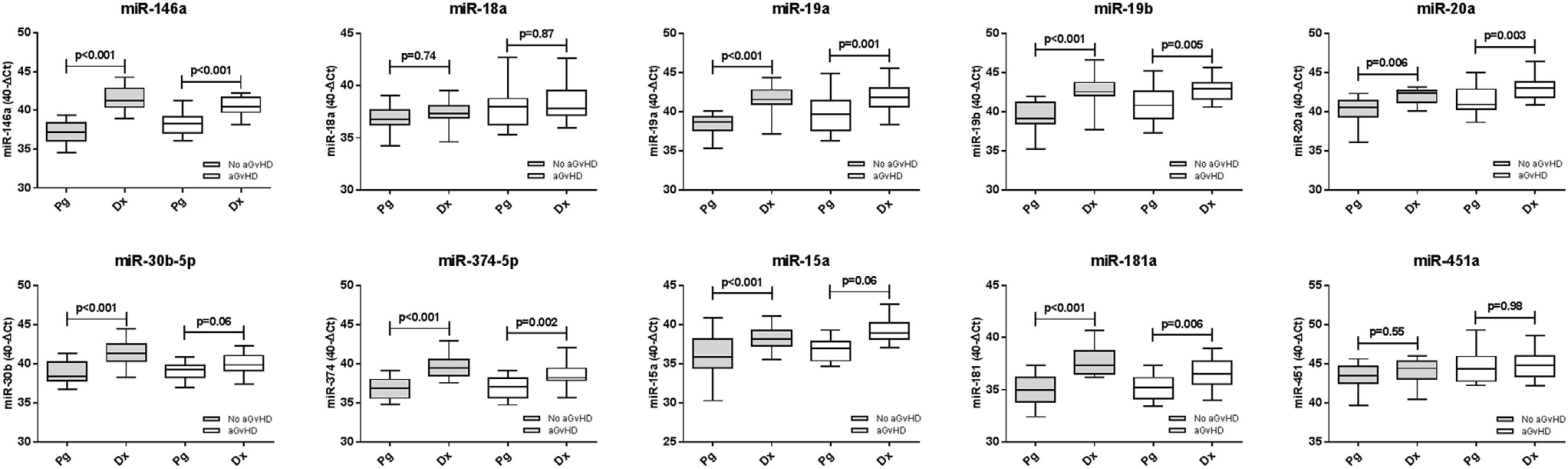
**Differential microRNA expression between prognostic and diagnostic samples**. Expression of the candidate microRNAs was compared between the diagnostic cohort (*n* = 42) and the day 14 prognostic cohort (*n* = 47) and analyzed according to acute graft-versus-host disease incidence. Box plot whiskers represent minimum to maximum expression, and *p* values were calculated between groups using the independent two sample *t*-test.

Expression of candidate microRNAs was compared between the time of onset samples of the diagnostic cohort (*n* = 42) and earlier D14 time points of the prognostic cohort (*n* = 47), according to aGvHD incidence. For both aGvHD and no aGvHD groups, expression levels of all microRNAs were significantly higher in the diagnostic cohort compared to the D14 prognostic cohort (*p* = < 0.001 to 0.006), except for miR-18a and miR-451a (no significant increase, *p* = 0.55–0.98) and miR-30b-5p and miR-15a (no significant increase in the aGvHD patient group, *p* = 0.06) (Figure 6).

### Pathway Analysis of Differential microRNAs at aGvHD Diagnosis

Candidate microRNAs identified by NanoString analysis were assessed for identification of potential target genes. This is a complex undertaking, due to the short nature of seed sequence regions being complementary to multiple target genes. Thus, Ingenuity Pathway Analysis was used to identify genes and classify targets into networks and pathways. Results indicated direct or indirect involvement of 15 of 61 microRNAs in the “cancer, hematological disease, immunological disease” pathway, including miR-146a and miR-18a of the verification microRNAs, 14 of 61 in the “organismal injury and inflammatory disease” pathway, including miR-181a and miR-19b of the verification microRNAs and 11 of 61 in the “cancer, connective tissue disorder, organismal injury and abnormalities” pathway, including miR-451a of the verification microRNAs (Figure S2 in Supplementary Material). With respect to diseases and functions, 5 of 61 microRNAs were associated with inflammatory disease (miR-18a, miR-17-5p, miR-146a, miR-19b, and miR-92a), whereas 19 of 61 were involved in the inflammatory response (miR-181, miR-185-5p, miR-18a, miR-193a-3p, miR-199, miR-19b, miR-30c, miR-320b, miR-425-5p, miR-513a, miR-92a, let-7a-5p, miR-100a-5p, miR-124-3p, miR-146, and miR-1285-3p). With regard to inflammatory response-associated microRNAs, the gene targets *SP1, FOS, DDX20, AGO2, TP53, RB1, E2F1-3, PTEN, MOV10, MYC*, and *ZEBZ* were experimentally validated or highly predicted.

## Discussion

Despite aGvHD being the most frequent and serious complication of HSCT, there are still no prognostic or diagnostic molecular biomarkers that are routinely used in the clinic to inform on treatment decisions and patients outcome. This may, in part, be due to an incomplete understanding of the molecular biology of the disease, which has precluded more personalized approaches to conditioning and prophylaxis regimens. Seminal research has previously identified blood proteins that associate with clinical outcome, including albumin, IL-2 receptor-α, tumor necrosis factor receptor 1, hepatocyte growth factor, IL-8, elafin, and REG-3α (33–37). However, these biomarkers cannot differentiate between GvHD and other inflammatory conditions, and thus, additional circulating molecules that may further improve upon the accuracy and efficiency of biomarker panels are required. This study sought to globally profile the expression of microRNAs at aGvHD diagnosis in the serum of post-HSCT patients, to identify microRNAs that demonstrate differential expression at the onset of disease symptoms. This focus on circulating microRNAs will allow for an understanding of molecules that may be biologically active at aGvHD diagnosis, as well as further assessed for their biomarker potential.

Although previous groups have performed microRNA profiling post-HSCT, these studies have focused on specific subgroups of patients or a refined signature of microRNAs limited by the qRT-PCR technology used (13, 17). This study was based on NanoString technology, which incorporates over 700 validated microRNA targets allowing for the most comprehensive assessment of circulating microRNA expression in a HSCT setting to date. Results identified 61 microRNAs that were differentially expressed between patients with aGvHD compared to those who remained disease free. Of these, 10 were selected for verification using qRT-PCR in independent cohorts of samples taken at diagnosis and prior to onset of symptoms (day 14 post-HSCT), based on their high FC values or prior association with aGvHD or the immune or inflammatory response.

In the diagnostic verification cohort (*n* = 42), miR-146a, miR-30b-5p, miR-374-5p, and miR-181 were significantly downregulated at aGvHD diagnosis compared to patients who remained aGvHD free. miR-146a is one of the more widely studied microRNAs with respect to aGvHD. Stickel et al. reported its upregulation in T-cells of mice developing aGvHD compared to untreated controls, and transplantation of miR-146a -/- T-cells resulted in increased aGvHD severity and reduced survival (31). In this study, miR-146a expression was significantly higher at aGvHD diagnosis compared to day 14 samples. Interestingly, Stickel et al. observed downregulated miR-146a shortly following allo-HCT in mice (day 2), followed by upregulation in T-cells later in the aGvHD reaction (days 6 and 12), which they hypothesized may be a rescue mechanism to counteract inflammation (31). As most microRNAs assessed in this study were expressed at a lower level following HSCT, it may be hypothesized that their translation is disrupted by the HSCT procedure, and this warrants further investigation. miR-30b-5p has been implicated in the acute allograft rejection process, whereby expression was downregulated in acute rejection (38), which is in keeping with the lower levels of miR-30b-5p observed in aGvHD in this study. Little is known about the role of miR-30b-5p in relation to HSCT, although it has been shown to be present in regulatory T-cells (Tregs), where it targets eight signaling pathways associated with their function (39). Expression of miR-374 has been identified in non-activated natural Tregs (nTregs), and there is evidence to suggest that nTregs may prevent allograft rejection (40). In this context, it may be surprising that miR-374 levels are lower at aGvHD diagnosis; however, nTregs comprise just 5–10% of the circulating CD4^+^ population, and pro-inflammatory cytokines released during the acute response can suppress nTreg function (41). miR-181 acts as an intrinsic modulator of T-cell sensitivity and selection (26), and in accordance with the present results showing lower miR-181 expression at aGvHD diagnosis, BMT-recipient mice transplanted with miR-181 negative donor T-cells demonstrate accelerated aGvHD (42).

In contrast, in the diagnostic verification cohort, expression of miR-20a, miR-18a, miR-19a, miR-19b, and miR-451 was significantly upregulated in aGvHD patients. These microRNAs were selected for verification as they are members of the miR-17-92 cluster, a well characterized loci that plays a fundamental role in T-cell differentiation, survival, and function as well as autoimmune responses (43–45). In relation to aGvHD, the cluster promotes CD4^+^ T-cell activation and Th1 differentiation, while inhibiting Th2 and iTreg differentiation and also promoting migration of CD8 T-cells to aGvHD target organs (32). Blockade of miR-17 or miR-19b inhibits alloreactive T-cell expansion and IFN-γ production and prolonged survival in GvHD afflicted mice (32). Thus, our data are in keeping with an aGvHD-promoting role for the cluster, whereby expression of its members was significantly upregulated at aGvHD diagnosis. Interestingly, the same microRNAs were also upregulated prior to symptomatic disease (at day 14), further supporting their role in T-cell response to alloantigens (32). Although the function of miR-451 is less well characterized, it has been shown to repress Myc expression (46) and indirectly inhibit the PI3K/AKT pathway (47), which regulates several key events in the inflammatory response to damage (48).

In addition to the microRNAs selected in this study for further verification, previous studies have associated other microRNAs with aGvHD. In a seminal study in 2012, Ranganathan et al. showed that miR-155 is upregulated in T-cells from mice developing GvHD following HSCT and the use of miR-155 inhibitors decreased disease severity and prolonged survival (49); however, this study focused on microRNA expression in tissue. Indeed, although Xiao et al. detected serum upregulation of miR-155 in aGvHD patients, expression levels were the lowest among the microRNA candidates examined, and thus, miR-155 was excluded from their final aGvHD signature (13). Xie et al. detected upregulation of miR-155 in aGvHD patient serum, which also correlated with disease severity; however, this study was restricted to a Han Chinese population (*n* = 64) (14). Collectively, the findings may highlight the heterogeneous nature of microRNA expression that is both tissue and condition specific. Thus, despite promising results in tissue (49), this may not be reflective of miR-155 expression in the circulation.

A small study by Sang et al. additionally reported upregulation of miR-92b in serum samples at aGvHD onset, while miR-150 and miR-181 were significantly downregulated (15). Expression was also altered prior to symptomatic disease, indicating potential biomarker potential for predicting disease incidence (15). The difference in miR-181 expression was most pronounced and also associated with disease severity (15). In this present study, miR-181 was also significantly downregulated in aGvHD compared to patients who remained aGvHD free (FC −2.26, *p* = 0.046). miR-181a has been shown to enhance CD4^+^ T lymphocytes toward Th2 and Treg differentiation, by targeting IFN-γ, and expression levels correlate with IFN-γ protein, but not mRNA expression (15). Further in-depth analysis of miR-181a expression, including expansion to functional studies, will be important to define the role of this microRNA in the aGvHD reaction.

With regard to microRNA profiling in a HSCT setting, in 2013 in an elegant study, Xiao et al. investigated the expression of 345 microRNAs in the plasma of patients with aGvHD, compared to patients with no aGvHD, using a qRT-PCR array (13). The study focused on a discovery cohort followed by a training cohort and identified a final signature of four microRNAs (miR-423, miR-199-3p, miR-93*, and miR-377) that significantly predicted for aGvHD at 6 weeks post-HSCT, prior to the onset of symptoms. Furthermore, the model was associated with disease severity and poor OS (13). This study highlighted the potential of biofluid microRNAs as independent markers for prediction, prognosis, and diagnosis of GvHD. Gimondi et al. also profiled circulating microRNA expression using a qRT-PCR-based platform (17). They assessed samples collected 28 days post-HSCT and detected 113 microRNAs, of which 27 could collectively discriminate between aGvHD vs. no aGvHD. miR-194 and miR-518f were significantly upregulated in patients who later developed aGvHD, and pathway prediction analysis identified these microRNAs to target critical pathways implicated in aGvHD pathogenesis (17). However, there was no verification cohort included in the investigation, and the authors did not detect differential expression of the microRNAs previously reported by Xiao et al. (13). Indeed, although expression of the Xiao et al. microRNA signature was disparate between aGvHD and no aGvHD groups in this study, this did not reach significance. When comparing the 27 unique microRNAs formerly identified by Gimondi et al., 7 (miR-374b/20a/185/191/30b) were also significantly associated with aGvHD in this study, while no statistically significant overlap was identified for the remaining microRNAs. The lack of reproducibility between microRNA profiling studies to date may not be surprising, due to the high degree of variability in factors when designing and performing these experiments, which may be attributed to clinical (patient characteristics, sampling time points, and type of body fluid analyzed), technical (sample preparation, microRNA profiling platform, and spectrum of microRNAs profiled), and analytical (normalization strategy) factors.

With respect to clinical characteristics, the patients included in this study comprised a mixed population of underlying disease, treatment prophylaxis, and conditioning regimen to reflect the diverse population of HSCT patients who develop aGvHD. Xiao et al. used a similar approach (13); however, the patients in the study by Gimondi et al. were restricted to lymphoma patients in complete remission undergoing RIC (17). In addition, different time points of sampling were used in these studies, and Xiao et al. investigated plasma taken 6 weeks posttransplant (10) and Gimondi et al. focused on day +28 posttransplant (17). Furthermore, although earlier studies concentrated on plasma (13, 17), serum was assessed in this study. It has been suggested that because serum is free of anticoagulants such as heparin, a potent inhibitor of downstream PCR reactions, it may favor more consistent measurements (50). In addition, serum processing is less likely to be affected by hemolysis than plasma, where the release of red blood cell and platelet-specific microRNAs during processing and may bias results.

In relation to technical considerations, substantial variability can be attributed to the RNA extraction method and microRNA profiling technology employed. A spin-column-based approach was used in this study; however, it was not dependent on phenol-based steps used in the previous studies (10, 24), which have been associated with low RNA purity leading to reproducibility issues, as well as selective loss of small RNA molecules with low GC content (51). The choice of microRNA detection platform is particularly important when distinguishing small molecules at low abundance and with high sequence homology (52). Xiao et al. based their assessment on Sybr-green dye, which lacks specificity in comparison to the TaqMan low-density array employed by Gimondi et al. (17). However, qRT-PCR is still reliant on reverse transcription of RNA to cDNA, which can introduce bias. This study relied on NanoString profiling, which is based on direct hybridization of reporter molecules to RNA, and thus removes the need for reverse transcription. The analysis is based on a direct digital count of tagged labeled barcodes, eradicating any background noise. Indeed NanoString technology has been shown to have greater consistency and a lower risk of introducing technical variation due to fewer sample preparation steps in comparison to qRT-PCR (53). Finally, the qRT-PCR arrays previously employed were restricted to a refined number of target molecules [*n* = 345 (13) and *n* = 377 (17)], and the degree of overlap between targets assessed in both studies is not clear. In contrast, miRNAs included in the nCounter Human v3 miRNA panel (*n* = 799) account for greater than 95% of all observed sequencing reads in miRBase v21, thus providing a far broader spectrum of microRNAs assessed that is not biased by preprofiling target selection.

Finally, analytical variables have the potential to bias results, and this is particularly dependent on the normalization methods used (54). Some level of correction may be achieved by spiking in synthetic microRNAs (55), which was the approach used by Xiao et al. (13); however, this method does not account for intrinsic biological variation (56). While Gimondi et al. calculated reciprocal ratios of the microRNA expression levels (17), this study incorporated 14 positive and negative controls, 6 ligation controls, 5 mRNA housekeeping controls, and a normalization factor based on the geometric mean of 100 targets with the highest counts.

In conclusion, although results implicate circulating microRNAs in the pathology of aGvHD, and their utility as disease biomarkers shows great promise, these studies are still in their infancy, and few overlapping targets between reports have been identified. Much work is needed to validate the findings in independent cohorts that fully reflect the high level of heterogeneity in conditioning and prophylaxis regimens employed by different clinical centers. This may be achieved through collaboration between research groups, focusing on standardization of the samples, protocols, and technologies used, which will greatly improve the reproducibility of findings allowing for extended validation of microRNAs of interest. The ultimate aim will be to diagnose GvHD and predict outcome before the onset of clinical symptoms, allowing for earlier therapy and personalized treatments and leading to reduced mortality and morbidity outcomes.

## Author Contributions

RC designed and performed the experiments, evaluated the data, and wrote the manuscript; JN assisted with verification experiments and drafting of the manuscript; MJ assisted with sample selection and data collection; KG advised on bioinformatics analysis of NanoString data; KP advised and performed statistical analysis; CL performed clinical data collection and advised on statistical analysis; HG contributed to clinical samples, data collection, and preparation of the manuscript; AD developed the overall concept, supervised the research, and prepared the manuscript. All authors approved the final manuscript.

## Conflict of Interest Statement

The authors declare that the research was conducted in the absence of any commercial or financial relationships that could be construed as a potential conflict of interest.

## Footnotes

^1^www.EuroTransplantBank.org.

^2^www.ingenuity.com.

## References

[1] Passweg JR Baldomero H Bader P Bonini C Cesaro S Dreger P Hematopoietic stem cell transplantation in Europe 2014: more than 40 000 transplants annually. Bone Marrow Transplant (2016) 51:786–92. 10.1038/bmt.2016.20 26901709 PMC4895175

[2] Reddy P Ferrara JL. Immunobiology of acute graft-versus-host disease. Blood Rev (2003) 17:187–94. 10.1016/S0268-960X(03)00009-2 14556773

[3] Chen X Ba Y Ma L Cai X Yin Y Wang K Characterization of microRNAs in serum: a novel class of biomarkers for diagnosis of cancer and other diseases. Cell Res (2008) 18:997–1006. 10.1038/cr.2008.282 18766170

[4] Mitchell PS Parkin RK Kroh EM Fritz BR Wyman SK Pogosova-Agadjanyan EL Circulating microRNAs as stable blood-based markers for cancer detection. Proc Natl Acad Sci U S A (2008) 105:10513–8. 10.1073/pnas.0804549105 18663219 PMC2492472

[5] Xi C Yi B Lijia M Xing C Yuan Y Kehui W Characterization of microRNAs in serum: a novel class of biomarkers for diagnosis of cancer and other diseases. Cell Res (2008) 18:997–1006. 10.1038/cr.2008.282 18766170

[6] Etheridge A Lee I Hood L Galas D Wang K. Extracellular microRNA: a new source of biomarkers. Mutat Res (2011) 717:85–90. 10.1016/j.mrfmmm.2011.03.004 21402084 PMC3199035

[7] Turchinovich A Weiz L Langheinz A Burwinkel B. Characterization of extracellular circulating microRNA. Nucleic Acids Res (2011) 39:7223–33. 10.1093/nar/gkr254 21609964 PMC3167594

[8] Vickers KC Palmisano BT Shoucri BM Shamburek RD Remaley AT. microRNAs are transported in plasma and delivered to recipient cells by high-density lipoproteins. Nat Cell Biol (2011) 13:423–33. 10.1038/ncb2210 21423178 PMC3074610

[9] Taylor DD Gercel-Taylor C. microRNA signatures of tumor-derived exosomes as diagnostic biomarkers of ovarian cancer. Gynecol Oncol (2008) 110:13–21. 10.1016/j.ygyno.2008.04.033 18589210

[10] Gallo A Tandon M Alevizos I Illei GG. The majority of microRNAs detectable in serum and saliva is concentrated in exosomes. PLoS One (2012) 7:e30679. 10.1371/journal.pone.0030679 22427800 PMC3302865

[11] Paczesny S. Discovery and validation of graft-versus-host disease biomarkers. Blood (2013) 121:585–94. 10.1182/blood-2012-08-355990 23165480 PMC3557644

[12] Wang K Yuan Y Cho J-H Mcclarty S Baxter D Galas DJ. Comparing the microRNA spectrum between serum and plasma. PLoS One (2012) 7:e41561. 10.1371/journal.pone.0041561 22859996 PMC3409228

[13] Xiao B Wang Y Li W Baker M Guo J Corbet K Plasma microRNA signature as a noninvasive biomarker for acute graft-versus-host disease. Blood (2013) 122:3365–75. 10.1182/blood-2013-06-510586 24041574 PMC3821726

[14] Xie LN Zhou F Liu XM Fang Y Yu Z Song NX Serum microRNA155 is increased in patients with acute graft-versus-host disease. Clin Transplant (2014) 28:314–23. 10.1111/ctr.12314 24494749

[15] Sang W Zhang C Zhang D Wang Y Sun C Niu M microRNA-181a, a potential diagnosis marker, alleviates acute graft versus host disease by regulating IFN-gamma production. Am J Hematol (2015) 90:998–1007. 10.1002/ajh.24136 26223969 PMC4801322

[16] Wang Y Zhao X Ye X Luo H Zhao T Diao Y Plasma microRNA-586 is a new biomarker for acute graft-versus-host disease. Ann Hematol (2015) 94:1505–14. 10.1007/s00277-015-2414-z 26051902

[17] Gimondi S Dugo M Vendramin A Bermema A Biancon G Cavane A Circulating miRNA panel for prediction of acute graft-versus-host disease in lymphoma patients undergoing matched unrelated hematopoietic stem cell transplantation. Exp Hematol (2016) 44:624–34.e. 10.1016/j.exphem.2016.03.005 27013207

[18] Glucksberg H Storb R Fefer A Buckner CD Neiman PE Clift RA Clinical manifestations of graft-versus-host disease in human recipients of marrow from HL-A-matched sibling donors. Transplantation (1974) 18:295–304. 10.1097/00007890-197410000-00001 4153799

[19] Filipovich AH Weisdorf D Pavletic S Socie G Wingard JR Lee SJ National Institutes of Health consensus development project on criteria for clinical trials in chronic graft-versus-host disease: I. Diagnosis and staging working group report. Biol Blood Marrow Transplant (2005) 11:945–56. 10.1016/j.bbmt.2005.09.004 16338616

[20] Crossland RE Norden J Bibby LA Davis J Dickinson AM Evaluation of optimal extracellular vesicle small RNA isolation and qRT-PCR normalisation for serum and urine. J Immunol Methods (2015) 429:39–49. 10.1016/j.jim.2015.12.011 26723490

[21] McNeil BJ Keeler E Adelstein SJ. Primer on certain elements of medical decision making. N Engl J Med (1975) 293:211–5. 10.1056/NEJM197507312930501 806804

[22] Fine JP Gray RJ A proportional hazards model for the subdistribution of a competing risk. J Am Stat Assoc (1999) 94:496–509. 10.1080/01621459.1999.10474144

[23] Iacobelli S EBMT Statistical Committee. Suggestions on the use of statistical methodologies in studies of the European group for blood and marrow transplantation. Bone Marrow Transplant (2013) 48(Suppl 1):S1–37. 10.1038/bmt.2012.282 23462821

[24] Gray RJ A class of K-sample tests for comparing the cumulative incidence of a competing risk. Ann Stat (1988) 16:1141–54. 10.1214/aos/1176350951

[25] Holm S A simple sequentially rejective multiple test procedure. Scand J Stat (1979) 6:65–70.

[26] Li QJ Chau J Ebert PJ Sylvester G Min H Liu G miR-181a is an intrinsic modulator of T cell sensitivity and selection. Cell (2007) 129:147–61. 10.1016/j.cell.2007.03.008 17382377

[27] Pan X Wang R Wang ZX. The potential role of miR-451 in cancer diagnosis, prognosis, and therapy. Mol Cancer Ther (2013) 12:1153–62. 10.1158/1535-7163.MCT-12-0802 23814177

[28] Su X Qian C Zhang Q Hou J Gu Y Han Y miRNomes of haematopoietic stem cells and dendritic cells identify miR-30b as a regulator of Notch1. Nat Commun (2013) 4:2903. 10.1038/ncomms3903 24309499 PMC3863901

[29] Williams A Henao-Mejia J Harman CC Flavell RA miR-181 and metabolic regulation in the immune system. Cold Spring Harb Symp Quant Biol (2013) 78:223–30. 10.1101/sqb.2013.78.020024 24163395

[30] Liu X Robinson SN Setoyama T Tung SS D’abundo L Shah MY FOXP3 is a direct target of miR15a/16 in umbilical cord blood regulatory T cells. Bone Marrow Transplant (2014) 49:793–9. 10.1038/bmt.2014.57 24710569 PMC4080423

[31] Stickel N Prinz G Pfeifer D Hasselblatt P Schmitt-Graeff A Follo M miR-146a regulates the TRAF6/TNF-axis in donor T cells during GVHD. Blood (2014) 124:2586–95. 10.1182/blood-2014-04-569046 25205119

[32] Wu Y Heinrichs J Bastian D Fu J Nguyen H Schutt S microRNA-17-92 controls T-cell responses in graft-versus-host disease and leukemia relapse in mice. Blood (2015) 126:1314–23. 10.1182/blood-2015-02-627356 26138686 PMC4566810

[33] Ferrara JL Harris AC Greenson JK Braun TM Holler E Teshima T Regenerating islet-derived 3-alpha is a biomarker of gastrointestinal graft-versus-host disease. Blood (2011) 118:6702–8. 10.1182/blood-2011-08-375006 21979939 PMC3242723

[34] Rezvani AR Storer BE Storb RF Mielcarek M Maloney DG Sandmaier BM Decreased serum albumin as a biomarker for severe acute graft-versus-host disease after reduced-intensity allogeneic hematopoietic cell transplantation. Biol Blood Marrow Transplant (2011) 17:1594–601. 10.1016/j.bbmt.2011.07.021 21806949 PMC3203323

[35] Harris AC Ferrara JL Braun TM Holler E Teshima T Levine JE Plasma biomarkers of lower gastrointestinal and liver acute GVHD. Blood (2012) 119:2960–3. 10.1182/blood-2011-10-387357 22286196 PMC3327467

[36] Levine JE Logan BR Wu J Alousi AM Bolanos-Meade J Ferrara JL Acute graft-versus-host disease biomarkers measured during therapy can predict treatment outcomes: a blood and marrow transplant clinical trials network study. Blood (2012) 119:3854–60. 10.1182/blood-2012-01-403063 22383800 PMC3335389

[37] Ayuk F Bussmann L Zabelina T Veit R Alchalby H Wolschke C Serum albumin level predicts survival of patients with gastrointestinal acute graft-versus-host disease after allogeneic stem cell transplantation. Ann Hematol (2014) 93:855–61. 10.1007/s00277-013-1957-0 24248672

[38] Anglicheau D Sharma VK Ding R Hummel A Snopkowski C Dadhania D microRNA expression profiles predictive of human renal allograft status. Proc Natl Acad Sci U S A (2009) 106:5330–5. 10.1073/pnas.0813121106 19289845 PMC2663998

[39] Chen L Ma H Hu H Gao L Wang X Ma J Special role of Foxp3 for the specifically altered microRNAs in regulatory T cells of HCC patients. BMC Cancer (2014) 14:489. 10.1186/1471-2407-14-489 25000974 PMC4099493

[40] Graca L Thompson S Lin CY Adams E Cobbold SP Waldmann H. Both CD4(+)CD25(+) and CD4(+)CD25(-) regulatory cells mediate dominant transplantation tolerance. J Immunol (2002) 168:5558–65. 10.4049/jimmunol.168.11.5558 12023351

[41] Valencia X Stephens G Goldbach-Mansky R Wilson M Shevach EM Lipsky PE. TNF downmodulates the function of human CD4+CD25hi T-regulatory cells. Blood (2006) 108:253–61. 10.1182/blood-2005-11-4567 16537805 PMC1895836

[42] Lee CW Wohlan K Dallmann I Forster R Ganser A Krueger A miR-181a expression in donor T cells modulates graft-versus-host disease after allogeneic bone marrow transplantation. J Immunol (2016) 196:3927–34. 27009493 10.4049/jimmunol.1502152

[43] Jiang S Li C Olive V Lykken E Feng F Sevilla J Molecular dissection of the miR-17-92 cluster’s critical dual roles in promoting Th1 responses and preventing inducible Treg differentiation. Blood (2011) 118:5487–97. 10.1182/blood-2011-05-355644 21972292 PMC3217351

[44] Khan AA Penny LA Yuzefpolskiy Y Sarkar S Kalia V. microRNA-17~92 regulates effector and memory CD8 T-cell fates by modulating proliferation in response to infections. Blood (2013) 121:4473–83. 10.1182/blood-2012-06-435412 23596046

[45] Liu SQ Jiang S Li C Zhang B Li QJ miR-17-92 cluster targets phosphatase and tensin homology and Ikaros Family Zinc Finger 4 to promote TH17-mediated inflammation. J Biol Chem (2014) 289:12446–56. 10.1074/jbc.M114.550723 24644282 PMC4007439

[46] Li X Sanda T Look AT Novina CD Von Boehmer H. Repression of tumor suppressor miR-451 is essential for NOTCH1-induced oncogenesis in T-ALL. J Exp Med (2011) 208:663–75. 10.1084/jem.20102384 21464222 PMC3135352

[47] Tian Y Nan Y Han L Zhang A Wang G Jia Z microRNA miR-451 downregulates the PI3K/AKT pathway through CAB39 in human glioma. Int J Oncol (2012) 40:1105–12. 10.3892/ijo.2011.1306 22179124 PMC3584578

[48] Hawkins PT Stephens LR. PI3K signalling in inflammation. Biochim Biophys Acta (2015) 1851:882–97. 10.1016/j.bbalip.2014.12.006 25514767

[49] Ranganathan P Heaphy CE Costinean S Stauffer N Na C Hamadani M Regulation of acute graft-versus-host disease by microRNA-155. Blood (2012) 119:4786–97. 10.1182/blood-2011-10-387522 22408260 PMC3367879

[50] Li Y Kowdley KV. Method for microRNA isolation from clinical serum samples. Anal Biochem (2012) 431:69–75. 10.1016/j.ab.2012.09.007 22982505 PMC3481852

[51] El-Khoury V Pierson S Kaoma T Bernardin F Berchem G. Assessing cellular and circulating miRNA recovery: the impact of the RNA isolation method and the quantity of input material. Sci Rep (2016) 6:19529. 10.1038/srep19529 26787294 PMC4726450

[52] Mestdagh P Hartmann N Baeriswyl L Andreasen D Bernard N Chen C Evaluation of quantitative miRNA expression platforms in the microRNA quality control (miRQC) study. Nat Methods (2014) 11:809–15. 10.1038/nmeth.3014 24973947

[53] Prokopec SD Watson JD Waggott DM Smith AB Wu AH Okey AB Systematic evaluation of medium-throughput mRNA abundance platforms. RNA (2013) 19:51–62. 10.1261/rna.034710.112 23169800 PMC3527726

[54] Leidner RS Li L Thompson CL. Dampening enthusiasm for circulating microRNA in breast cancer. PLoS One (2013) 8:e57841. 10.1371/journal.pone.0057841 23472110 PMC3589476

[55] Sapre N Selth LA. Circulating microRNAs as biomarkers of prostate cancer: the state of play. Prostate Cancer (2013) 2013:10. 10.1155/2013/539680 23577261 PMC3610368

[56] Huang X Liang M Dittmar R Wang L. Extracellular microRNAs in urologic malignancies: chances and challenges. Int J Mol Sci (2013) 14:14785–99. 10.3390/ijms140714785 23863690 PMC3742273

