# Supplementary material for: Expression of Serum microRNAs is Altered During Acute Graft-versus-Host Disease
Source: Front Immunol. 2017 Mar 24;8:308. doi: 10.3389/fimmu.2017.00308 (PMC5364146; doi:10.3389/fimmu.2017.00308)

Supplementary Figure 1. MiR-146a expression in normal healthy controls. Expression of miR-146a was assessed by qRT-PCR in 3 independent healthy controls, in triplicate repeats, across 3 consecutive days (time points 1-3). The Ct expression values are shown and p-values were calculated by one-way ANOVA.

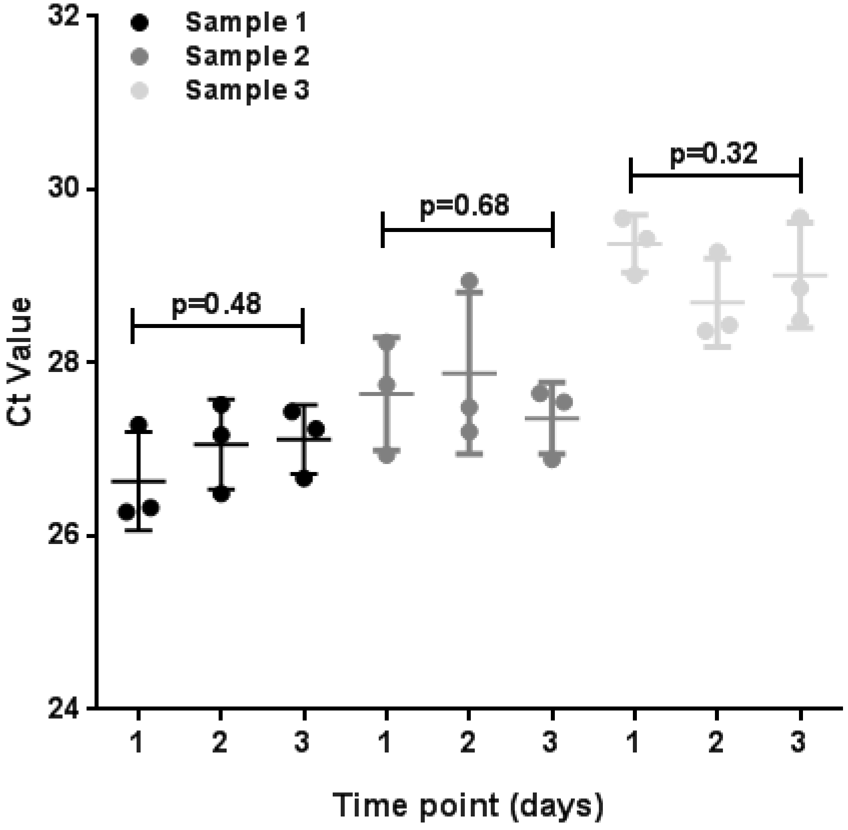

Supplement: Supplementary file 1 [file image_1.pdf]
