# Supplementary material for: Expression of Serum microRNAs is Altered During Acute Graft-versus-Host Disease
Source: Front Immunol. 2017 Mar 24;8:308. doi: 10.3389/fimmu.2017.00308 (PMC5364146; doi:10.3389/fimmu.2017.00308)
Supplement: Supplementary file 3 [file table_1.pdf]

Supplementary Table 1. Inter-assay and intra-assay variation for qRT-PCR data. An example microRNA (miR-15a) was assessed for inter-assay and intra-assay variation. Expression was measured for 3 independent samples, repeated on 3 independent days, in triplicate. Individual Ct values are shown, as well as the mean, standard deviation (SD) and coefficient of variance (CV) for each repeat. Pooled inter- and intra-assay CV values were calculated using the root mean square method.

Sample	Repeat	Triplicate Ct	Ct Mean	Ct SD	Ct CV
1	1	27.82<br>28.24<br>27.56	27.88	0.34	0.0123
	2	27.69<br>27.20<br>26.05	26.98	0.84	0.031
	3	27.86<br>28.01<br>27.42	27.76	0.31	0.0110
2	1	25.69<br>25.71<br>24.47	25.29	0.71	0.0282
	2	25.86<br>25.47<br>25.34	25.56	0.27	0.010
	3	24.98<br>25.32<br>25.76	25.35	0.39	0.0154
3	1	25.08<br>24.86<br>26.22	25.39	0.73	0.0287
	2	26.07<br>25.18<br>24.45	25.23	0.81	0.0321
	3	25.69<br>24.81<br>25.54	25.35	0.47	0.0186
**Pooled CV**		**Inter-assay**		1.89%	
		**Intra-assay**		2.26%	
