# Supplementary material for: Expression of Serum microRNAs is Altered During Acute Graft-versus-Host Disease
Source: Front Immunol. 2017 Mar 24;8:308. doi: 10.3389/fimmu.2017.00308 (PMC5364146; doi:10.3389/fimmu.2017.00308)
Supplement: Supplementary file 4 [file table_2.pdf]

Supplementary Table 2. NanoString-detected fold changes in microRNA quantity between aGvHD versus no aGvHD patients. 11 allo-HSCT patient serum samples (6 aGvHD, 5 no aGvHD) were included in the final dataset for microRNA expression assessment (n=799) using the NanoString microRNA panel. Table indicates the relationship between fold change and significance between aGvHD compared to no aGvHD patients.

Gene Name	Accession Number	Fold Change	p-Value
hsa-miR-302b-3p	MIMAT0000715	5.41	0.004387
hsa-miR-1910-5p	MIMAT0007884	4.36	0.006463
hsa-miR-320c	MIMAT0005793	3.85	0.031232
hsa-miR-193b-3p	MIMAT0002819	3.78	0.007977
hsa-miR-506-3p	MIMAT0002878	3.43	0.003282
hsa-miR-451a	MIMAT0001631	3.20	0.001550
hsa-miR-486-3p	MIMAT0004762	3.17	0.011411
hsa-miR-514a-5p	MIMAT0022702	3.08	0.008668
hsa-miR-7-5p	MIMAT0000252	2.83	0.000243
hsa-miR-18a-5p	MIMAT0000072	2.82	0.030423
hsa-miR-503-3p	MIMAT0022925	2.80	0.005541
hsa-miR-1285-3p	MIMAT0005876	2.75	0.009938
hsa-miR-425-5p	MIMAT0003393	2.63	0.012834
hsa-miR-3074-3p	MIMAT0015027	2.62	0.033319
hsa-miR-93-5p	MIMAT0000093	2.50	0.001004
hsa-miR-892a	MIMAT0004907	2.49	0.015542
hsa-miR-191-5p	MIMAT0000440	2.36	0.004763
hsa-miR-491-5p	MIMAT0002807	2.32	0.019328
hsa-miR-513a-5p	MIMAT0002877	2.29	0.001370
hsa-miR-25-3p	MIMAT0000081	2.28	0.005098
hsa-miR-1299	MIMAT0005887	2.22	0.011887
hsa-miR-19a-3p	MIMAT0000073	2.10	0.006983
hsa-miR-19b-3p	MIMAT0000074	2.06	0.008910
hsa-miR-185-5p	MIMAT0000455	2.05	0.045984
hsa-miR-450a-5p	MIMAT0001545	1.96	0.006050
hsa-miR-20a-5p+20b-5p	MIMAT0000075	1.90	0.012073
hsa-miR-513b-5p	MIMAT0005788	1.90	0.019586
hsa-miR-1183	MIMAT0005828	1.79	0.017629
hsa-miR-140-5p	MIMAT0000431	1.79	0.039147
hsa-miR-520a-5p	MIMAT0002833	1.77	0.005510
hsa-let-7b-5p	MIMAT0000063	1.69	0.011503
hsa-miR-520d-3p	MIMAT0002856	1.64	0.039421
hsa-miR-148b-3p	MIMAT0000759	1.62	0.016190
hsa-miR-15a-5p	MIMAT0000068	1.35	0.024642
hsa-miR-548ah-5p	MIMAT0018972	-1.75	0.029469
hsa-miR-378b	MIMAT0014999	-1.80	0.030142
hsa-miR-199a-5p	MIMAT0000231	-1.83	0.043925
hsa-miR-1246	MIMAT0005898	-1.90	0.006117
hsa-miR-607	MIMAT0003275	-2.05	0.016157
hsa-miR-4286	MIMAT0016916	-2.10	0.046583
hsa-miR-149-5p	MIMAT0000450	-2.15	0.048417
hsa-miR-181a-3p	MIMAT0000270	-2.26	0.046447
hsa-miR-495-5p	MIMAT0022924	-2.27	0.045465
hsa-miR-4531	MIMAT0019070	-2.79	0.029445
hsa-miR-424-5p	MIMAT0001341	-2.86	0.021711
hsa-miR-100-5p	MIMAT0000098	-2.95	0.020711
hsa-miR-515-3p	MIMAT0002827	-3.03	0.043717
hsa-miR-1290	MIMAT0005880	-3.07	0.022028
hsa-miR-1270	MIMAT0005924	-3.10	0.036672
hsa-miR-376c-3p	MIMAT0000720	-3.18	0.029953
hsa-miR-3185	MIMAT0015065	-3.21	0.000875
hsa-let-7f-5p	MIMAT0000067	-3.25	0.000418
hsa-miR-1206	MIMAT0005870	-3.26	0.038259
hsa-miR-30b-5p	MIMAT0000420	-3.39	0.036526
hsa-miR-138-5p	MIMAT0000430	-3.41	0.021995
hsa-miR-146a-5p	MIMAT0000449	-4.53	0.010073
hsa-miR-141-3p	MIMAT0000432	-4.82	0.003522
hsa-miR-6721-5p	MIMAT0025852	-4.91	0.017809
hsa-miR-374b-5p	MIMAT0004955	-5.02	0.011036
hsa-miR-1915-3p	MIMAT0007892	-6.17	0.033179
hsa-miR-556-5p	MIMAT0003220	-6.94	0.040513
