# Supplementary material for: Expression of Serum microRNAs is Altered During Acute Graft-versus-Host Disease
Source: Front Immunol. 2017 Mar 24;8:308. doi: 10.3389/fimmu.2017.00308 (PMC5364146; doi:10.3389/fimmu.2017.00308)
Supplement: Supplementary file 5 [file table_3.pdf]

Supplementary Table 3. Correlation analysis between candidate validation microRNAs. Expression of microRNAs in the diagnostic validation cohort (n=42) was assessed by Pearson correlation. P-values and correlation coefficients are shown (p-value:r value). P-values (<0.05) are highlighted in bold. *Indicates significance following Holm-Bonferonni sequential multiple comparisons correction.

	**miR-146a**	**miR-18a**	**miR-19a**	**miR-19b**	**miR-20a**	**miR-30b**	**miR-374**	**miR-15a**	**miR-181**	**miR-451**
**miR-146a**		0.955: 0.010	0.315: 0.167	0.099: 0.272	0.320: 0.168	**0.000*: 0.814**	**0.000*: 0.782**	**0.001*: 0.532**	**0.000*: 0.775**	0.700: -0.071
**miR-18a**	0.955: 0.010		**0.000*: 0.810**	**0.000*: 0.780**	**0.000*: 0.700**	0.155: 0.235	0.081: 0.291	**0.025: 0.374**	0.391: 0.150	**0.000*: 0.785**
**miR-19a**	0.315: 0.167	**0.000*: 0.810**		**0.000*: 0.972**	**0.000*: 0.663**	**0.004: 0.452**	**0.014: 0.401**	**0.001*: 0.529**	**0.044: 0.343**	**0.000*: 0.852**
**miR-19b**	0.099: 0.272	**0.000*: 0.780**	**0.000*: 0.972**		**0.002: 0.479**	**0.005: 0.454**	**0.011: 0.412**	**0.000*: 0.608**	**0.009: 0.428**	**0.000*: 0.844**
**miR-20a**	0.320: 0.168	**0.000*: 0.700**	**0.000*: 0.663**	**0.002: 0.479**		0.368: 0.154	0.125: 0.261	0.386: 0.151	0.360: 0.159	**0.000*: 0.760**
**miR-30b**	**0.000*: 0.814**	0.155: 0.235	**0.004: 0.452**	**0.005: 0.454**	0.368: 0.154		**0.000*: 0.963**	**0.000*: 0.700**	**0.000*: 0.804**	0.342: 0.174
**miR-374**	**0.000*: 0.782**	0.081: 0.291	**0.014: 0.401**	**0.011: 0.412**	0.125: 0.261	**0.000*: 0.963**		**0.000*: 0.792**	**0.000*: 0.863**	0.309: 0.189
**miR-15a**	**0.001*: 0.532**	**0.025: 0.374**	**0.001*: 0.529**	**0.000*: 0.608**	0.386: 0.151	**0.000*: 0.700**	**0.000*: 0.792**		**0.000*: 0.875**	**0.016: 0.430**
**miR-181**	**0.000*: 0.775**	0.391: 0.150	**0.044: 0.343**	**0.009: 0.428**	0.360: 0.159	**0.000*: 0.804**	**0.000*: 0.863**	**0.000*: 0.875**		0.110: 0.288
**miR-451**	0.700: -0.071	**0.000*: 0.785**	**0.000*: 0.852**	**0.000*: 0.844**	**0.000*: 0.760**	0.342: 0.174	0.309: 0.189	**0.016: 0.430**	0.110: 0.288	
