# Supplementary material for: Expression of Serum microRNAs is Altered During Acute Graft-versus-Host Disease
Source: Front Immunol. 2017 Mar 24;8:308. doi: 10.3389/fimmu.2017.00308 (PMC5364146; doi:10.3389/fimmu.2017.00308)

Supplementary Figure 2. Pathway and network analysis for NanoString significant genes. The 61 significant genes identified by NanoString analysis were profiled using Ingenuity Pathway Analysis (IPA) for associated pathways and networks. Graphical representations of networks were based on direct and indirect targets. (A) Cancer, haematological disease and immunological disease network. Score 32, focus molecules 15. (B) Organismal injury and inflammatory disease network. Score 30, focus molecules 14. (C) Cancer, connective tissue disorder, organismal injury and abnormalities network. Score 22, focus molecules 11. Red = up-regulation; green = downregulation.

Cancer, haematological disease, immunological disease | **Organismal injury and inflammatory disease** | **Cancer, connective tissue disorder, organismal injury and abnormalities**

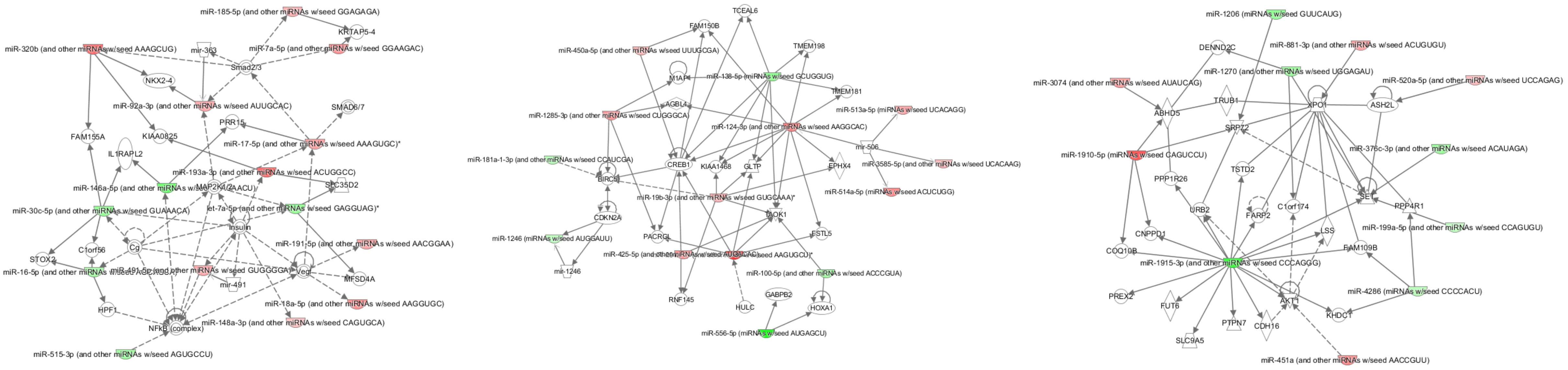

Supplement: Supplementary file 2 [file image_2.pdf]
